# The Swedish RAND-36 Health Survey - reliability and responsiveness assessed in patient populations using Svensson’s method for paired ordinal data

**DOI:** 10.1186/s41687-018-0030-0

**Published:** 2018-02-07

**Authors:** Lotti Orwelius, Mats Nilsson, Evalill Nilsson, Marika Wenemark, Ulla Walfridsson, Mats Lundström, Charles Taft, Bo Palaszewski, Margareta Kristenson

**Affiliations:** 1 Department of Anaesthesiology and Intensive Care, and Department of Clinical and Experimental Medicine, Linköping University, Linköping, Sweden; 2 Futurum, - Academy for Health and Care, Region Jönköping County, Jönköping, Sweden; 3 QRC Stockholm Research Unit, Medical Management Centre, Department of Learning, Informatics, Management and Ethics, Karolinska Institutet, Stockholm, Sweden; 4 Department of Medical and Health Sciences, Faculty of Medicine, Linköping University, Linköping, Sweden; 5 Centre for Organisational support and Development, Region Östergötland, Linköping, Sweden; 6 Department of Cardiology, and Department of Medical and Health Sciences, Faculty of Medicine, Linköping University, Linköping, Sweden; 7 Department of Clinical Sciences, Ophthalmology, Faculty of Medicine, Lund University, Lund, Sweden; 8 Centre of registers, Västra Götaland, Göteborg, Sweden; 9 Data Management and Analysis, Region Västra Götaland, Göteborg, Sweden; 10 Intensive Care Unit, University Hospital, Linkoping, Sweden

**Keywords:** Psychometrics, Validation, SF-36, Health-related quality of life, Patient-reported outcome measure, Translations

## Abstract

**Background:**

The Short Form 36-Item Survey is one of the most commonly used instruments for assessing health-related quality of life. Two identical versions of the original instrument are currently available: the public domain, license free RAND-36 and the commercial SF-36.

RAND-36 is not available in Swedish. The purpose of this study was threefold: to translate and culturally adapt the RAND-36 into Swedish; to evaluate its reliability and responsiveness using Svensson’s method for paired ordered categorical data; and to assess the usability of an electronic version of the questionnaire.

The translation process included forward and backward translations and reconciliation. Test-retest reliability was examined during a period of two-weeks in 84 patients undergoing dialysis for chronic kidney disease. Responsiveness was examined in 97 patients before and 2 months after a cardiac rehabilitation program. Usability tests and cognitive debriefing of the electronic questionnaire were carried out with 18 patients.

**Results:**

The Swedish translation of the RAND-36 was conceptually equivalent to the English version. Test-retest reliability was supported by non-significant relative position (RP) values among dialysis patients for all RAND-36 subscales (range − 0.02 to 0.10; all confidence intervals (CI) included zero). Responsiveness was demonstrated by significant improvements in RP values among cardiac rehabilitation patients for all subscales (range 0.22–0.36; lower limits of all CI > 0.1) except two subscales (General health, RP -0.02; CI -0.13 to 0.10; and Role functioning/emotional, RP 0.03; CI -0.09 to 0.16). In cardiac rehabilitation patients, sizable individual variation (RV > 0.2) was also shown for the Pain, Energy/fatigue and Social functioning subscales.

The electronic version of RAND-36 was found easy and intuitive to use.

**Conclusions:**

Our results provide evidence supporting the reliability and responsiveness of the newly translated Swedish RAND-36 and the user-friendliness of the electronic version. Svensson’s method for paired ordinal data was able to characterize not only the direction and size of differences among the patients’ responses at different time points but also variations in response patterns within groups. The method is therefore, besides being suitable for ordinal data, also an important and novel tool for gaining insights into patients’ response patterns to treatment or interventions, thus informing individualized care.

## Background

Patient-reported outcome measures (PROMs) are important and clinically relevant tools for evaluating treatment and rehabilitation outcomes from a patient perspective [1]. In Sweden, the National Quality Registers (NQRs), today numbering over 100, are currently encouraged to include PROMs in their arsenal of outcome measures and are required to collect PROM data to attain highest levels of registry classification [2, 3].

One of the most commonly used generic measures of health-related quality of life (HRQoL) is the Short Form 36-Item Survey version 1.0, developed in the RAND Medical Outcomes Study during the 1980s [4]. Two identical versions of the questionnaire are currently available: the RAND-36 Item Health Survey [5], a public domain form, and the SF-36 Item Health Survey [6], a copyrighted, commercially distributed form. Minor differences exist between RAND-36 and SF-36 in scoring procedures for two of the eight subscales and the RAND-36 lacks an authorized algorithm for calculating Mental and Physical Component Summary scores. The SF-36 (where a license and a license fee is required for usage) has been available in Swedish since the early 1990s [7]; however, lately there has been requests for a Swedish version of the public domain RAND-36. Therefore, work to translate and culturally adapt the RAND-36 to contemporary Swedish and to develop an electronic version of the questionnaire was initiated.

RAND-36, like most questionnaires, generates ordinal (ordered categorical) level data for each item, which are in turn aggregated to subscale scores. Such scores may be correctly treated as a new ordinal scale, or treated as an approximation of an interval or ratio level scale although such an approximation may lead to misleading conclusions [8–10]. Appropriate methods for analyzing ordinal data are available. One such method is Svensson’s method for paired ordinal data [11, 12], which is suitable for both reliability and responsiveness analyses and also enables analysis of individual variation.

The aims of this multicenter study were to translate RAND-36 into Swedish and evaluate its reliability and responsiveness using Svensson’s method for paired ordinal data. Another aim was to assess the usability of an electronic version of the questionnaire.

## Methods

### RAND-36

The RAND-36 is a 36-item questionnaire intended for use as a generic measure of HRQoL (https://www.rand.org/health/surveys_tools/mos/36-item-short-form.html). Using the standard scoring algorithm from RAND Corporation, eight conceptual attributes (subscales) are calculated by averaging values of 35 of the 36 ordinal scale items. The remaining item (Health change), assesses change in perceived health during the last year. Subscale scores range from 0 to 100, where higher scores represent better health status.

### Translation process

The aim was to develop a conceptually equivalent translation written in contemporary Swedish. The translation process included: two forward translations performed independently by two native Swedish speaking, certified translators from a professional translation agency (TransPerfect Ltd., NY, USA); reconciliation of the forward translations and cultural adaptations by an expert review panel; a back translation from Swedish to English by a native English speaking, certified translator; and finally a reconciliation of the final version based on results of the back translation [13]. Discrepancies or problems in the translation were resolved by discussions between the translator, back-translator and the expert review panel. The panel consisted of researchers experienced in questionnaire development and PROMs with special insight into respondents’ difficulties in responding to the SF-36 [14]. A special feature in this translation process is the fact that a Swedish version of the SF-36 already exists, translated in cooperation with the creator of SF-36, John Ware, and culturally adapted using IQOLA methodology [7]. Although the original English versions of SF-36 and RAND-36 are identical, a new translation is bound to differ from an existing translation. To ensure that these differences did not impact on the content validity of the questionnaire, comparisons with the Swedish version of the SF-36 were made throughout the translation process.

### Evaluation of reliability and responsiveness

#### Participants

Inclusion criteria were age ≥ 18 years, able to read and understand Swedish and to complete the questionnaire independently. Patients were consecutively included during the study period. Patients gave their consent to participate orally and by answering the questionnaire after having received written and oral study information.

#### Dialysis patients for testing reliability

Test-retest reliability was assessed in patients with chronic kidney disease undergoing dialysis, as their condition is expected to be clinically stable during a test–retest period of two-weeks [15]. Patients requiring dialyses for diagnoses such as glomerulonephritis or diabetic nephropathy (inclusion criterion) were recruited from five clinics at four hospitals. Dialyses included hemo or peritoneal dialysis, performed at hospital or at home either with assistance or alone. Only patients who completed their retest questionnaire within a period of 7–17 days after the first one were included in data analyses.

#### Cardiac rehabilitation patients for testing responsiveness

Responsiveness was assessed in patients with ischemic heart disease participating in a cardiac rehabilitation program after an cardiac event as their condition is expected to improve over a period of 2–3 months [15]. Included patients had had an acute myocardial infarction and/or undergone a percutaneous coronary intervention and/or coronary artery bypass surgery for unstable angina due to ischemic heart disease (inclusion criterion). Patients were recruited from six clinics at six different hospitals. The rehabilitation program varied between clinics and was performed individually or in groups. Patients who completed follow-up questionnaires within a period of 50 to 70 days after the first one were included in the data analyses.

#### Measurements and procedures

Baseline questionnaires included the RAND-36 and a set of background questions on age, sex, educational status, employment, height and weight and physician-diagnosed comorbidities. At retest/follow-up, the questionnaire contained only the RAND-36.

Questionnaires were handed out during visits at the clinic and answered at the time of the visit or sent home to the patient. Patients who answered at home could either hand in the questionnaire at the next planned visit or send it back to the clinic in an enclosed pre-paid envelope. The healthcare professionals who administered the questionnaires were instructed not to assist the patients in completing the questionnaire or to check for unanswered items since it was a validation study.

#### Statistics, general

RAND subscale scores may be computed even when all items are not answered, i.e. with partially missing items [5, 11]; however, in this paper, subscales with item-nonresponse were excluded from the analysis, since missing data and/or imputed values may introduce bias in the estimates [16].

Internal consistency was calculated using the ordinal alpha method [17–19] instead of the traditional Cronbach’s alpha method. The former is based on polychoric correlations and assumes continuity in the underlying construct, not that data themselves are continuous, whereas the latter is based on Pearson correlations and assumes that data are continuous. Ordinal alpha has the same limits for acceptable internal consistency as Cronbach and an alpha of > 0.90 is often recommended for instruments intended for use at an individual level [15]. A SAS®/IML macro was used to calculate ordinal alpha [20].

#### Specific statistics- Svensson’s method

Svensson’s method for analyzing agreement in paired ordinal data was used to study test-retest reliability (hypothesis: no change in the dialysis group) and responsiveness (hypothesis: a positive change, improvement, in the cardiac rehabilitation group). The method is described in detail elsewhere [8]. Analysis software with an instruction manual and interpretation guide are available for download [11].

##### Percentage agreement (PA)

The proportion of identical answers at two measurement points.

##### Relative position (RP)

The degree of systematic change, either improvement or deterioration, in variable values between two measurement points. The cumulative frequency (marginal distribution) of variable values is illustrated in a Receiver Operating Characteristic (ROC) curve, where a bow-shaped ROC curve indicates a systematic change in position of variable values.

Numerically, RP is calculated as the difference between the probability of improvement and the probability of deterioration (range + 1 to - 1). For example, if the probability is 0.70 that higher values occur at retest/follow-up than at baseline (improvement) and the probability is 0.27 that higher values occur at baseline than at retest/follow-up (deterioration), the RP value will be 0.70–0.27 = 0.43 (RP = 0.43), i.e. 43% units greater probability for improvement than for deterioration.

##### Relative concentration (RC)

Systematic shift in the concentration of ratings to the centre of the rating scale at different measurement points (seen in the ROC analyses as an S-shaped curve). For this, the RC is computed analogously with RP as a difference between two probabilities, where a positive value indicates that answers are more concentrated in the center at retest/follow-up, and a negative value means that they are more concentrated at baseline (range − 1 to + 1).

##### Relative rank variance (RV)

Estimate of individual variability in ranks between two measurement points (range 0 to 1). Higher values on RV (at least > 0.20, according to Svensson) are an indication of individual departures from a common pattern of change; i.e. RV is a measure of heterogeneity in relation to the expected group change. In most empirical cases, some individual variation is expected alongside any systematic changes of the groups.

RP, RC and RV are presented with standard errors and 95% confidence intervals (if the interval includes zero, there is no significant change in RP or RC).

### Design, usability testing and cognitive debriefing of the electronic version

The electronic version was designed to resemble the paper-and-pencil version as closely as possible. The main difference is that the electronic version displays 3–5 items per screen, whereas all 36 items are presented on two pages in the paper-and-pencil version. Additional instructions explaining how to respond were added to the electronic version. As in the paper version, it is possible to skip single items. Though evidence suggests that such minor changes will not affect the performance of a questionnaire, it is still advisable to test the questionnaire on a small sample of respondents [21].

A stratified purposeful sample representing different age groups, levels of computer literacy, and diagnoses was chosen among patients at four clinics that had specifically requested an electronic version Patients included those undergoing ambulatory care for kidney disease, cancer patients active in patient organizations, patients referred for catheter ablation treatment due to arrhythmia, and patients recently (2 months) discharged from intensive care. The first two patient groups responded to the electronic questionnaire using a computer, and the latter two using a tablet. In total, ten men and eight women aged 35–77 years were invited to participate and all agreed.

The interviews were conducted by four different interviewers. The interviewer first observed the respondents as they completed the questionnaire, and clocked the completion time. Then they performed semi-structured interviews regarding the respondents’ experiences of answering the questionnaire, any problems encountered, readability of the text, navigating the questionnaire, etc.

## Results

### Translation process

The translation of colloquial expressions and common daily physical activities were to a certain extent aligned with the existing Swedish SF-36. Well-known problems with the SF-36/RAND-36 (including the Swedish version of SF-36), such as the double negation in item 19 “Didn’t do work or other activities as carefully as usual”, which has been rectified in SF-36 version 2, were also rectified in the new Swedish RAND-36. Daily activities used to exemplify certain items were chosen and adapted to represent activities that are common in Sweden today. For example, in the item about moderate physical activities “moving a table, pushing a vacuum cleaner, bowling, or playing golf” was changed to “moving a table, pushing a vacuum cleaner, walking, or cycling”.. The expert review panel concluded that the new translation was conceptually equivalent to the original instrument, since it contained no content differences compared with the current Swedish SF-36. Differences between the Swedish versions of the RAND-36 and the SF-36 concerned language updates.

### Evaluation of reliability and responsiveness

A total of 213 dialysis patients and 360 cardiac rehabilitation patients were invited to participate in the study. Of those, 204 (95%) dialysis patients and 268 (74%) cardiac rehabilitation patients accepted the invitation. In total, 169 (83%) dialysis patients and 223 (83%) cardiac rehabilitation patients responded at both occasions. However, only 84 (41%) and 97 (36%), respectively, responded within the stipulated time periods (reliability 7–17 days; responsiveness 50–70 days).

The number of patients who answered all items on each subscale varied between 71 and 83 (out of 84) and 86–97 (out of 97) for each subscale (Table 1). No single item or subscale was especially exposed to item nonresponse.

**Table 1:** Number (percent) of patients with no item-missing by subscale and patient group

Subscale (no. of items) item numbers (1–36)	Patient group	
	Dialysis patients (*n* = 84)	Cardiac rehabilitation patients (*n* = 97)
	n (%)	n (%)
Physical functioning (10) 3, 4, 5, 6, 7, 8, 9, 10, 11, 12	72 (86%)	88 (91%)
Role functioning/physical (4) 13, 14, 15, 16	71 (85%)	82 (85%)
Pain (2) 21, 22	76 (90%)	94 (97%)
General health (5) 1, 33, 34, 35, 36	75 (89%)	90 (93%)
Energy/fatigue (4) 23, 27, 29, 31	77 (92%)	88 (91%)
Social functioning (2) 20, 32	79 (94%)	97 (100%)
Role functioning/emotional (3) 17, 18, 19	71 (85%)	86 (89%)
Emotional well-being (5) 24, 25, 26, 28, 30	79 (94%)	92 (95%)
Health change (1) 2	83 (99%)	92 (95%)

Table 2 presents sociodemographic characteristics for the two patient samples. As expected, the majority of patients were male, above 65 years of age and had multiple morbidities, and no unexpected differences between the two groups were found (e.g. a higher percentage of men among cardiac rehabilitation patients was expected due to the higher incidence among men). The sociodemographic distribution corresponds to that of the Swedish population in this age group [22, 23].

**Table 2:** Age, sex, educational level, employment, and co-morbidity by patient group

	Dialysis patients N = 84	Cardiac rehabilitation patients *N* = 97
	n (%)	n (%)
Age		
≤44	8 (9%)	0 (0%)
45–64	19 (23%)	40 (41%)
≥65	57 (68%)	57 (59%)
Sex		
Female	35 (42%)	28 (29%)
Male	49 (58%)	69 (71%)
Educational level		
Nine-year compulsory school	37 (45%)	38 (39%)
Upper secondary school	31 (37%)	42 (43%)
College/ University	15 (18%)	17 (18%)
Employment		
Employed/self-employed	10 (12%)	27 (28%)
Sick leave	6 (7%)	5 (5%)
Retired	54 (65%)	58 (60%)
Other	13 (16%)	7 (7%)
Co-morbidity		
No disease	2 (2%)	6 (6%)
One disease	20 (24%)	27 (28%)
Two diseases	18 (21%)	29 (30%)
Three or more diseases	44 (52%)	35 (36%)

Note that not all percentages add to 100 due to rounding to nearest integer

#### Internal consistency and subscale scores

Ordinal coefficient alphas for each subscale are presented in Table 3. Alpha values were largely the same in both patient groups, so the table shows alphas for the combined samples. Alpha values varied between 0.86 and 0.97, i.e. the internal consistency was satisfactory. Means and 95% confidence intervals for subscale scores for the two patient groups are also presented in Table 3.

**Table 3:** Ordinal coefficient alpha and RAND-36 subscale scores for the two patient groups

RAND – 36 subscales	Ordinal α	Subscale scores Mean (95% CI)			
	Total population (*n* = 181)	Dialysis patients (n = 84)		Cardiac rehabilitation patients (n = 97)	
		Baseline	Retest	Baseline	Follow up
Physical functioning	0.97	46 (39–53)	47 (40–54)	61 (57–64)	73 (70–77)
Role functioning/ physical	0.97	24 (16–32)	30 (22–38)	27 (21–32)	48 (42–54)
Pain	0.93	57 (51–64)	62 (56–69)	54 (50–58)	74 (70–77)
General health	0.86	37 (32–41)	36 (32–41)	57 (55–60)	62 (59–65)
Energy/fatigue	0.89	48 (44–53)	48 (43–53)	51 (48–54)	64 (61–67)
Social functioning	0.89	61 (55–66)	58 (52–64)	63 (59–66)	77 (74–80)
Role functioning/ emotional	0.94	55 (45–65)	54 (44–63)	56 (50–62)	66 (60–71)
Emotional well-being	0.90	68 (63–73)	70 (65–75)	71 (68–73)	78 (75–80)

*CI* Confidential Interval

#### Reliability and responsiveness

The Health change item (item 2) measures self-reported change in health over the last year. With a few exceptions (see below), the results for all the other items were very similar to those for the health change item, and therefore we chose to show only this item in detail.

Table 4 shows that most dialysis patients had identical ratings on this question at baseline and retest (64% on the diagonal, i.e. yellow boxes). Allowing for one response scale step differences in ratings, percentage agreement was 88%. RP and RC were close to zero, indicating no change between time points (Table 6).

**Table 4:**
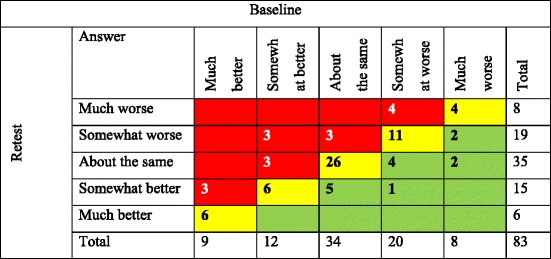
Test-retest reliability: Health change item ratings for dialysis patients at baseline and retest

The diagonal (yellow) represents patients who answered the same at baseline and retest (*n* = 53; PA = 64%), those who improved are shown below the diagonal (green) (*n* = 14, 17%) and those worsened are above the diagonal (red) (*n* = 16, 19%)

Table 5, on the other hand, reveals that many cardiac rehabilitation patients reported improved health at follow-up.

**Table 5:**
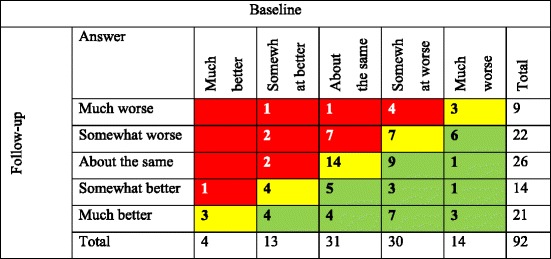
Responsiveness: Health change item ratings for cardiac rehabilitation patients at baseline and follow-up

The diagonal (yellow) represents patients who answered the same at baseline and follow-up (*n* = 31; PA = 34%), those who improved are shown below the diagonal (green) (*n* = 43, 46%) and those worsened are above the diagonal (red) (*n* = 18, 20%)

**Fig. 1:**
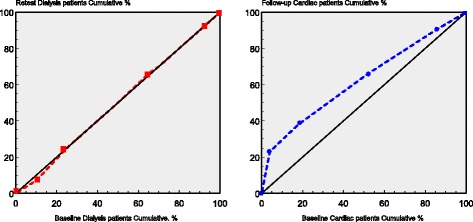
ROC-curve for dialysis patients and cardiac rehabilitation patients illustrating the change in the Health change item between the two measurements. The curves present the cumulative marginal distribution and show no differences for the dialysis patients, but an increase in patients with better health now than a year ago for the cardiac rehabilitation patients

The significant RP of 0.25 for the cardiac rehabilitation patients (Table 6) means that there is a 25 percentage unit higher probability that patients rated their health as better now than a year ago rather than as worse. RC showed that the responses from cardiac rehabilitation patients were more concentrated towards the middle response alternatives (“About the same” / “Somewhat worse”) at baseline than at follow-up, whereas dialysis patients showed no concentration changes. RV showed that the individual variation among cardiac patients was not negligible (also indicated by the RC), whereas dialysis patients showed only small individual variations. The ROC-curves (Fig. 1) for the dialysis patients (left) and cardiac rehabilitation patients (right) illustrate the results of the RP measurements. The curves present the cumulative distribution (in percent) of the two measurement points.

**Table 6:** Evaluation of change in the Health change item using Svensson’s method for the patient groups

Result	Dialysis patients (Reliability)				Cardiac rehabilitation patients (Responsiveness)			
PA	64%	SE	95% CI		34%	SE	95% CI	
RP	− 0.001	0.04	− 0.08	0.07	**0.25**	0.07	0.12	0.38
RC	−0.035	0.06	−0.15	0.08	**−0.18**	0.07	−0.32	− 0.04
RV	0.08	0.02	0.03	0.12	**0.34**	0.08	0.18	0.49

*SE* Standard Error, *CI* Confidential Interval, *PA* Percentage Agreement, *RP* Relative Position, *RC* Relative Concentration, *RV* Relative Rank Variation. Significant values are given in bold

The test-retest-analysis for the dialysis patients (Table 7) showed, as hypothesized, no significant changes in RP for any of the RAND-36 subscales. A few subscales had significant RC and RV values indicating that some individual change had occurred, although the group as a whole had not changed significantly.

**Table 7:** Test-retest analysis using Svensson’s method for ordinal paired data for the dialysis patients

RAND-36 subscale	PA	RP	SE	95% CI		RC	SE	95% CI		RV	SE	95% CI	
Physical functioning	33%	0.02	0.05	−0.07	0.12	− 0.02	0.04	− 0.11	0.06	**0.22**	0.11	0.01	0.42
Role functioning/ physical	51%	0.09	0.07	−0.04	0.22	0.08	0.07	−0.05	0.21	0.13	0.05	0.04	0.23
Pain	41%	0.08	0.05	−0.01	0.17	−0.06	0.07	−0.20	0.07	0.13	0.04	0.05	0.22
General health	20%	−0.05	0.05	−0.14	0.03	−0.07	0.07	−0.20	0.06	**0.21**	0.04	0.13	0.29
Energy/fatigue	10%	0.02	0.05	−0.07	0.11	−0.10	0.07	−0.23	0.04	0.16	0.04	0.07	0.24
Social functioning	30%	−0.05	0.06	−0.16	0.06	**−0.18**	0.07	−0.31	− 0.05	**0.22**	0.07	0.07	0.36
Role functioning/ emotional	63%	−0.01	0.04	−0.11	0.09	0.03	0.04	−0.05	0.12	0.09	0.10	0.00	0.29
Emotional well-being	23%	0.04	0.04	−0.03	0.12	−0.04	0.00	−0.15	0.05	0.13	0.04	0.04	0.21

*SE* Standard Error, *CI* Confidential Interval, *PA* Percentage Agreement, *RP* Relative Position (−1/+ 1), *RC* Relative Concentration, *(−1/+ 1) RV* Relative Rank Variation (0–1). Significant values are given in bold

The responsiveness analyses for the cardiac rehabilitation patients (Table 8) showed, as hypothesized, significant improvements in RP for all subscales except General health and Role functioning/emotional. Most subscales had significant RV and/or RC values indicating that some individual changes had occurred in addition to the systematic changes regarding RP.

**Table 8:** Responsiveness analysis using Svensson’s method for ordinal paired data for the Cardiac rehabilitation patients

RAND-36 subscales	PA	RP	SE	95% CI		RC	SE	95% CI		RV	SE	95% CI	
Physical functioning	13%	**0.31**	**0.05**	**0.21**	**0.41**	−0.15	0.08	−0.31	0.00	**0.28**	0.06	0.16	0.40
Role functioning/ physical	45%	**0.24**	**0.07**	**0.10**	**0.38**	**0.18**	**008**	**0.03**	**0.32**	**0.21**	0.06	0.08	0.33
Pain	19%	**0.30**	**0.06**	**0.17**	**0.42**	**0.17**	**0.09**	**0.01**	**0.34**	**0.41**	0.08	0.26	0.57
General health	19%	0.10	0.06	−0.01	0.21	−0.01	0.07	−0.14	0.13	**0.30**	0.08	0.15	0.45
Energy/fatigue	10%	**0.26**	**0.05**	**0.15**	**0.37**	0.00	0.08	−0.16	0.16	**0.26**	0.06	0.15	0.37
Social functioning	34%	**0.32**	**0.05**	**0.21**	**0.42**	0.02	0.08	−0.12	0.17	**0.23**	0.06	0.11	0.35
Role functioning/ emotional	51%	0.03	0.07	−0.10	0.16	0.05	0.06	−0.06	0.17	0.18	0.06	0.07	0.29
Emotional well-being	13%	**0.17**	**0.05**	**0.08**	**0.26**	0.04	0.07	−0.10	0.18	**0.22**	0.05	0.11	0.32

*SE* Standard Error, *CI* Confidential Interval, *PA* Percentage Agreement, *RP* Relative Position (−1/+ 1), *RC* Relative Concentration, *(−1/+ 1) RV* Relative Rank Variation (0–1). Significant values are given in bold

### Results of the testing of the electronic version

All 18 patients answered the questionnaire in three to 10 min except for two patients who needed 21 and 32 min, respectively (median 6 min). In general, the respondents found the electronic version easy to use (easy to navigate, read and select response alternatives), and only one person (an older person with limited experience of computers/tablets) stated that he/she would have preferred a traditional paper-and-pencil questionnaire. No problems were observed or reported when completing the questionnaire using a computer; however, tablets were generally more difficult to use by beginners, particularly when resizing text and scrolling. The interviews did not cover issues related to item content and yielded no new information about potential difficulties in completing the RAND-36.

## Discussion

This study reports on the translation and initial psychometric assessment of the Swedish RAND-36. Applying a novel method specifically designed for analysis of ordinal data, the study provides detailed evidence for the reliability and responsiveness of the Swedish RAND-36. The electronic version of RAND-36 was found easy and intuitive to use.

### Reliability and responsiveness

As hypothesized, test-retest reliability was generally supported in patients undergoing dialysis, as indicated by statistically non-significant changes in RP values, as was responsiveness in cardiac rehabilitation patients by statistically significant improvements in RP values. However, exceptions were found regarding the responsiveness of the subscales General Health and Role functioning/emotional.

Poor responsiveness of the General Health subscale has been reported in earlier studies, both in cardiac patients and other patient groups [24–26]. This subscale is composed of five items, of which two assess current health status and three items involve health comparisons with others and future health (easier to get sick than others, being as healthy as other people, and anticipation of deteriorating health). The latter three items may not be very responsive to changes over relatively short time periods, in fact only item 1 (the well-known global self-rated health item, known as SRH) showed significant changes in the present study. It might therefore be informative to consider item 1 on its own if the subscale is not responsive. Regarding Role functioning/emotional we do not have an obvious explanation-This scale does have a ceiling effect [7], and the cardiac rehabilitation groups do not address role-emotional issues specifically.

PROMs are increasingly used in evaluations of health care to demonstrate effects of new treatments and for health economic evaluations. The RAND-36 has rapidly attracted much attention in Sweden and several NQRs have already started to use it as their PROM of choice. Whilst this is very important, it is also important that such evaluations serve as a springboard for improving treatments and healthcare delivery [3]. In the present study the cardiac rehabilitation patients showed sizable individual variation (RV values > 0.20). Placing a greater emphasis on examining such variations in patients’ responses to treatment, to better understand why some but not all patients benefit from certain interventions, may be an important step in improving the quality of treatment and care. We have not found any studies that use methods to identify individual variation in patient outcomes in routine health services. We believe that Svensson’s method is an important tool that could help identify subgroups that do, or do not, benefit from treatment and hence lead the way to more individualized healthcare interventions.

### Methodological considerations

As has generally been the case in translating the SF-36 [12], relatively few difficulties were noted in translating items or response subscales of RAND-36 and generally the need to culturally adapt items was limited to replacing examples of daily activities common in the US with their equivalents in Sweden and substituting US colloquialisms with Swedish ones, in line with the existing Swedish version of SF-36. In an upcoming study, we will compare SF-36 and RAND-36 by means of differential item functioning analyses (Rasch analysis) to further ensure (concept) equivalence.

A possible concern in this study is that the final number of evaluable questionnaires was low. The main reason for this was that patients returned questionnaires after the stipulated time periods (17 days and 70 days, respectively). Late response generally owed to late mail back but was also due to logistical reasons, such as postponed revisits. However, there were no appreciable differences in background characteristics between those who responded within the time limits and those who responded late. Another factor possibly contributing to a smaller number of evaluable questionnaires was that the research staff was requested to not assist the patients or to check for and ask patients to fill in unanswered questions. In all analyses only questionnaire data with complete answers to all items comprising a subscale were analyzed to ensure that analyses were unbiased by missing values. However, as seen in Table 1, there was rather little partial missing data.

A possible disadvantage of the reduction of sample size is loss of power for psychometric analyses. However, Svensson’s method is found to be very robust and possible to use even in small study samples, with as few as ten to twelve subjects [27].

This study is unique in assessing reliability and responsiveness by means of a method specially developed for analyzing paired ordinal data, namely Svensson’s method [11]. This method is particularly suitable for ordinal questionnaire data as in the present study, and theoretically superior to several of the methods commonly used. Reliability is often estimated using Intraclass Correlation Coefficient (ICC) or Kappa analyses [15]. However, ICC theoretically requires data on at least interval-level, which is not the case with questionnaire data. Kappa analyses might also be problematic since they may underestimate agreement in some situations (e.g. if one response option is chosen much more often than all others) [15, 20]. For testing responsiveness, McNemar’s test is commonly used for paired ordered categorical data. However, McNemar’s test only informs if a change is significant or not, not the direction or the magnitude of the change. In addition to this kind of information, Svensson’s method also provides information about change on an individual level rather than just group-level change [9, 11]. This has the advantage of enabling the identification of subgroups with different profiles or responses to a certain treatment or intervention than the rest of the patient population.

In this study we regarded the subscale scores as ordinal level data and analyzed data using methods compatible with this level of measurement. However, when computing subscale scores we applied the RAND-36 standard algorithm whereby scores are computed as the mean of item ratings. Arguably, median values may be more appropriate for summarizing ordinal level items [8–10, and]; however, we found that our results were only marginally influenced by using mean or median based subscale values. The main differences were found to be lower PAs and higher RVs when using means instead of medians. This is expected given that the mean-based subscale scores have a larger number of possible score values, and hence exact agreement is more difficult to achieve. We chose to present only the analyses based on the standard scoring method; however, the pros and cons of using methods that acknowledge the ordinal nature of item data when calculating subscales merits further investigation and we will return to this topic and to comparisons between Svensson’s method and common parametric methods in general in future studies.

### The electronic version

The electronic version was designed to resemble the paper-and-pencil version as closely as possible, including the possibility to skip single items. Previous studies, in several different patient groups, for different ages and different health and computer literacy, etc., have revealed that electronic versions of RAND-36 and the SF-36 produce comparable data with the paper-and-pencil versions, supporting the use of mixed-mode administrations [28–32]. In the present study, one elderly respondent stated this person would have preferred to use a paper-and-pencil version. It has been shown that older people tend to prefer paper-and-pencil administrations (probably because of computer illiteracy, as in the present study), whereas younger people and people with higher education tend to prefer electronic versions [33]. The main difference between the electronic and the paper-and-pencil versions is that fewer items are displayed at the same time [34]. Earlier studies have shown that this in fact may impact responses, but also that many persons prefer to view only a few items at the same time since displays with many items can be perceived as stressful [30, 35]. Our results indicate that the electronic version was easy to understand but some minor adjustments to font size, line spacing, etc. may enhance readability. Tablet user instructions may need to be extended to address issues of resizing and scrolling. Computer literacy is high in Sweden, which means that the acceptability of the electronic version in countries with less computer-literate populations may be lower.

## Conclusions

The newly translated Swedish RAND-36 was found to be reliable and responsive in the two patient groups tested, i.e. patients undergoing dialysis and cardiac rehabilitation, respectively, and the electronic questionnaire was found to be a feasible surrogate for the paper-and-pencil version. Svensson’s method for paired ordinal data was able to characterize not only the direction and size of group differences among the patients’ responses at different time points but also individual variations in response patterns within groups. Svensson’s method is therefore, besides being a method developed for paired ordinal data, also an important and novel tool for evaluating individual response to treatment or interventions, thus informing individualized care.

## Abbreviations

HRQoL: Health-Related Quality of Life
ICC: Intraclass Correlation Coefficient
NQRs: National Quality Registers
PA: Percentage agreement
PROMs: Patient-reported outcome measures
RC: Relative concentration
ROC: Receiver Operating Characteristic
RP: Relative position
RV: Relative rank variance
SF-36: Medical Outcome Short-Form
